# Structures and growth pathways of Au_n_Cl_n+3_^-^ (n ≤ 7) cluster anions

**DOI:** 10.3389/fchem.2024.1382443

**Published:** 2024-03-11

**Authors:** Shiyin Xu, Xinhe Liu, Yameng Hou, Min Kou, Xinshi Xu, Filip Veljković, Suzana Veličković, Xianglei Kong

**Affiliations:** ^1^ State Key Laboratory of Elemento-Organic Chemistry, Frontiers Science Center for New Organic Matter, College of Chemistry, Nankai University, Tianjin, China; ^2^ ‘‘VINCA” Institute of Nuclear Sciences—National Institute of the Republic of Serbia, University of Belgrade, Belgrade, Serbia; ^3^ Tianjin Key Laboratory of Biosensing and Molecular Recognition, College of Chemistry, Nankai University, Tianjin, China

**Keywords:** gold chloride clusters, mass spectrometry, aurophilic interaction, growth pathway, structural optimization

## Abstract

Gold chloride clusters play an important role in catalysis and materials chemistry. Due to the diversity of their species and isomers, there is still a dearth of structural studies at the molecular level. In this work, anions of Au_n_Cl_n+3_^-^ and Au_n_Cl_n+5_^-^ (n = 2–4) clusters were obtained by laser desorption/ionization mass spectrometry (LDI MS), and the most stable isomers of Au_n_Cl_n+3_^-^ were determined after a thorough search and optimization at the TPSSh/aug-cc-pVTZ/ECP60MDF level. The results indicate that all isomers with the lowest energy have a planar zigzag skeleton. In each species, there is one Au(III) atom at the edge connected with four Cl atoms, which sets it from the other Au(I) atoms. Four growth pathways for Au_n_Cl_n+3_^-^ (n = 2–7) clusters are proposed (labelled R1, R2, R3 and R4). They are all associated with an aurophilic contact and are exothermic. The binding energies tend to stabilize at ∼ −41 kcal/mol when the size of the cluster increases in all pathways. The pathway R1, which connects all the most stable isomers of the respective clusters, is characterized by cluster growth due to aurophilic interactions at the terminal atom of Au(I) in the zigzag chains. In the pathway of R4 involving Au-Au bonding in its initial structures (n ≤ 3), the distance between intermediate gold atoms grows with cluster size, ultimately resulting in the transfer of the intermediate Au-Au bonding into aurophilic interaction. The size effect on the structure and aurophilic interactions of these clusters will be better understood based on these discoveries, potentially providing new insights into the active but elusive chemical species involved in the corresponding catalytic reactions or nanoparticle synthesis processes.

## 1 Introduction

Gold chemistry is one of the most rapidly developing chemical fields. The synthesis and growth mechanisms of gold nanoparticles are important topics that have attracted the attention of researchers in recent decades. The Brust-Schiffrin two-phase method, in which AuCl_4_ is reduced by sodium borohydride in the presence of an alkanethiol (Brust et al., 1994), is a widely used approach for the synthesis of small gold nanoparticles. Originally, it was assumed that the Au(I) thiolate polymers were formed as intermediates prior to the final reduction of the gold thiolate nanoparticles (Templeton et al., 2000; Murray, 2008; Sardar et al., 2009). However, Goulet and Lennox found that changing the ratio of thiol ligand to Au(III) salt from 4:1 to 2:1 did not lead to the formation of Au(I) thiolate oligomers, but to the reduction of Au(I)X_2_^-^ (X = halide), casting doubt for the first time that Au(I) thiolate oligomers are reaction intermediates (Goulet and Lennox, 2010). Research by Li *et al.* supports this view and suggests that metal nucleation centers form before metal-sulfur bonds are formed (Li et al., 2011). Barngrover *et al.* have shown that Au(I)X_2_^-^ (X = halide) is an important precursor for the synthesis of larger size gold nanoclusters (Barngrover et al., 2015).

Thanks to the above-mentioned works, gold halides and their corresponding clusters have become the subject of research in recent years (Davies et al., 2016; Cao et al., 2019; Zhang et al., 2020). For example, it is realized that the acetylene hydrochlorination over an Au/C catalyst involves the formation of Au-Cl related complexes (Davies et al., 2016), and so do the Au-catalyzed intramolecular hydroarylation reactions (Zhang et al., 2020). It should be noted that the properties of gold are influenced by relativistic effects (Theilacker et al., 2015; Jerabek et al., 2017), which is why the d^10^ electrons of the inner shell must also be taken into account when forming compounds and clusters. One of the most striking phenomena in this context is the aurophilic interaction. The short Au···Au distance observed in crystals is attributed to the weak interaction defined as “aurophilicity” or “aurophilic interaction” (Schmidbaur et al., 1988a; Schmidbaur et al., 1988b; Scherbaum et al., 1988). Aurophilic interactions bring the metal atoms closer together and contribute significantly to the stability of the corresponding clusters. Typically, the aurophilic interaction appears to act between closed-shell gold atoms and the Au^+1^ oxidation state. It is also characterized by the linear coordination of n = 2 of the gold atoms. To date, there are numerous studies that attempt to illustrate how aurophilicity can influence the structures of gold halide clusters. Schwerdtfeger *et al.* found that all coin metal(I) halide tetramers should have a square ring structure with a butterfly D_*2d*_ symmetry. However, gold(I) phosphane chloride and bromide tetramers have structures with planar gold cores due to gold-gold interactions (Schwerdtfeger et al., 2004). Rabilloud reported the lowest energy isomers of Au_n_Br_n_ and Au_n_I_n_ (n = 2–6). The cyclic isomers of tetramers and pentamers are non-planar to strengthen both the interactions between gold and halide atoms and those between neaby gold atoms (Rabilloud, 2012). Ma *et al.* have shown that all Au_n_Cl_n+1_^-^ (2 ≤ n ≤ 7) ions are characterized by a zigzag structure, suggesting that the aurophilic interaction plays a key role in such clusters (Ma et al., 2019b). Ma *et al.* also showed that Au_2n_Cl (n = 1–4) and Au_n_Cl_n+1_ (2 ≤ n ≤ 7) clusters can be experimentally obtained by matrix assisted laser desorption ionization mass spectrometry of AuCl_4_H (Ma et al., 2019a). Lemke investigated the formation of [AuCl_2-x_OH_x_]^+^(H_2_O)_n_ (x = 0.1), [AuCl_2_]^+^(HCl)_2_(H_2_O)_n_ and [Au_2_Cl_5-x_OH_x_]^+^(H_2_O)_n_ (x = 0.1) using electrospray ionization mass spectrometry and theoretical calculations. His calculations show that the microsolvation-induced localization of electrons enhances the Au···Au interaction in the singly bridged clusters (Lemke, 2014).

Because thiolate-for-chloride ligand exchange can happen at low barrier heights (Barngrover and Aikens, 2012), it is easy for the precursors of gold-chloride clusters or nanoparticles to form gold-chloride-thiolate or gold-thiolate clusters or nanoparticles by exchanging ligands. Therefore, understanding the structures of various series of gold chloride clusters, their growth pathways, and related kinetics is crucial for the growth of relevant gold nanoparticles. Despite the aforementioned studies on gold chloride clusters (Schwerdtfeger et al., 2004; Srivastava and Misra, 2014; Ma et al., 2019a; Ma et al., 2019b), there is still a gap in the study of polychlorinated gold cluster anions (for example, Au_n_Cl_n+3_^-^) and their growth pathways. In this paper, we attempt to reveal the stability, structural regularity and cluster growth patterns of Au_n_Cl_n+3_^-^ clusters by experimental methods and theoretical calculations. The most stable structures of the clusters and the relative binding properties within them as well as the trends of binding energy changes along the different growth paths are calculated and analyzed. The results are helpful in predicting relevant clusters with larger sizes and may also provide clues for the synthesis of one- or two-dimensional nanostructures from the unit of gold chloride.

## 2 Experimental

The experiments in this work were performed with a matrix-assisted laser desorption/ionization mass spectrometer, MALDI MS, (Voyager-DE PRO Sciex, Foster City, CA, United States) equipped with a time-of-flight (TOF) mass analyzer. This mass spectrometer contains an ultraviolet nitrogen laser (wavelength 337 nm, pulse width 3 ns and repetition rates of 20.00 Hz). The other instrumental parameters were: accelerating voltage 20,000 V, grid voltage 94%, laser intensity of 1,300 a. u., and number of laser shots 300 and delayed extraction time of 100 ns.

To avoid interference of matrix on cluster ions or potential unexpected reactions, the mass spectra were obtained using the laser desorption/ionization (LDI) method without the use of conventional or other matrices. The sample was aqueous AuCl_4_H solutions (2.5 g/dm^3^) (99.99%, CAS no.: 12453-07-1) prepared in deionized water (Millipore) immediately prior to the experiment. Subsequently, 0.5 μL of the AuCl_4_H solution was applied to the stainless target spot, the target spot left to dry at room temperature and placed in the source area of the mass spectrometer.

### 2.1 Computation

NKCS program (Zhou et al., 2021) has been applied here to search for global minimum structures of Au_n_Cl_n+3_^-^ (n = 2, 3, 4) clusters, and some other structures on the suggested growth pathways are constructed manually based on their structural similarity. All structures have been optimized by the Gaussian 09 program at the level of TPSSh with the Stuttgart energy-consistent relativistic pseudopotentials ECP60MDF and the corresponding valence basis set of MDF60 for Au and aug-cc-pVTZ for Cl. All structures are further checked by frequency calculations to make sure they are true energy minima in their potential energy surfaces (PESs). Besides, IRI (Interaction Region Indicator) (Lu and Chen, 2021), ELF (Electron Localization Function) (Lu and Chen, 2011) and AdNDP (Adaptive natural density partitioning) (Zubarev and Boldyrev, 2008) analysis have been carried out to investigate their bonding properties using Multiwfn program (Lu and Chen, 2012).

## 3 Results and discussion

The results of our previous studies have shown that the method of LDI mass spectrometry is suitable for the investigation of gold chloride clusters. For example, a series of Au_n_Cl_n+1_, Au_n_Cl_n-1_ and Au_n_Cl_n_H type cluster anions were obtained by laser ablation of AuCl_4_H (20 mg/mL) in the presence of graphene as a matrix. By reducing the AuCl_4_H concentration tenfold (2 mg/mL), the anions Au_2n_Cl^−^ and Au_2n+1_ (n = 1–4) could also be detected. A Nd:YAG laser (355 nm) was used for these experiments. The amount of sample applied to the target was 1 mL.

**FIGURE 1:**
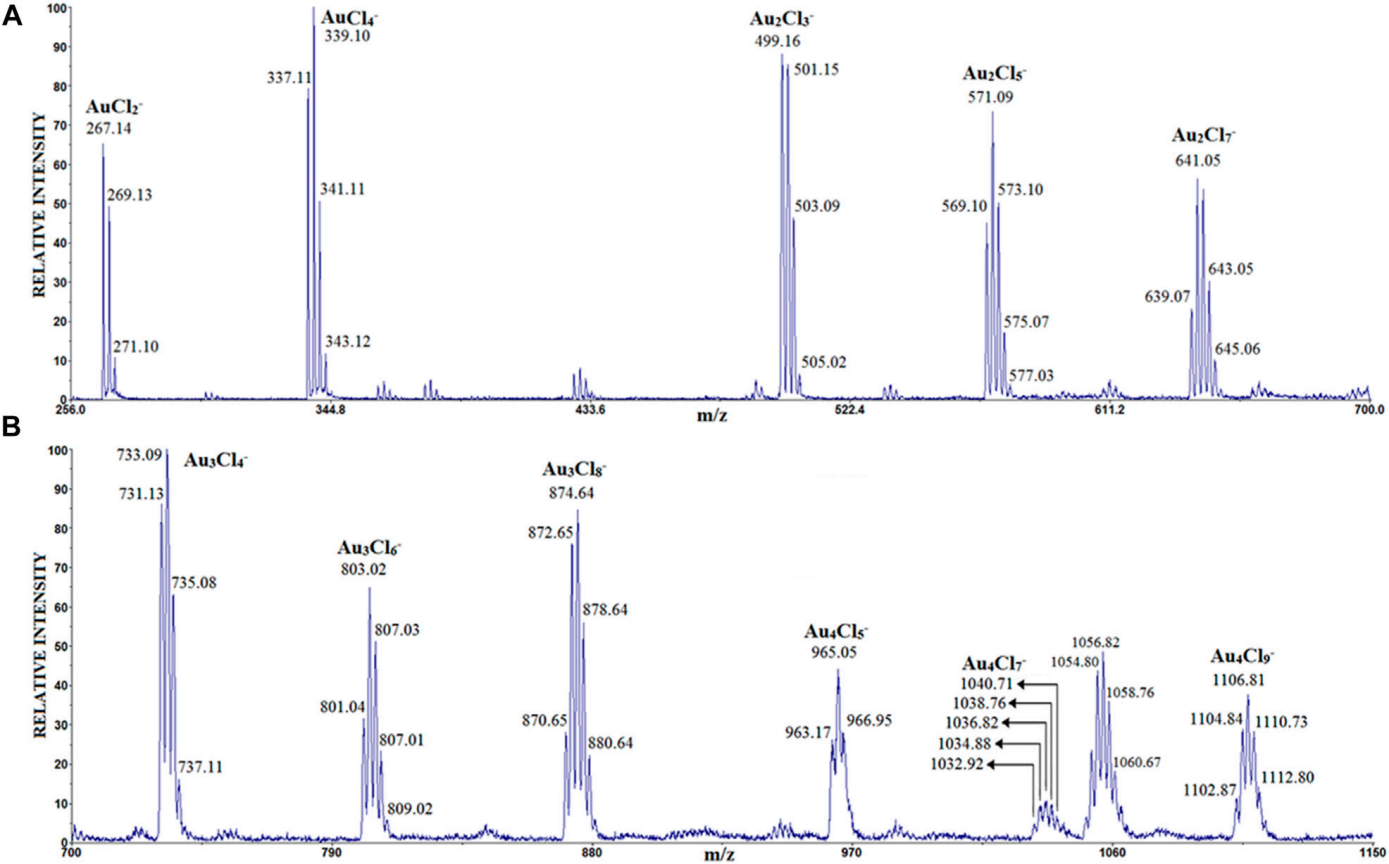
The LDI mass spectrum of AuCl_4_H obtained in the negative ion mode: **(A)** *m/z* 200–700 and **(B)** *m/z* 700–1,150). The attribution of peaks observed at ∼ m/z 1,056 that are differently spaced from the series of Au halide clusters (shown in b) needs further identification.

In contrast to the above conditions, here the amount of sample applied to the target was twice as small, desorption and ionization of the sample was performed with an N_2_ laser, mass spectra were obtained without using a matrix, while the concentration of AuCl_4_H was 2.5 mg/mL, similar to the previous experiments. Under these conditions, the anion clusters Au_n_Cl_n+1_^-^, (n = 1–4), Au_n_Cl_n+3_^-^ and Au_n_Cl_n+5_^-^ (n = 2, 3, 4) were identified in the LDI mass spectrum in negative mode, as shown in Figures 1A,B (for the sake of clarity, the mass spectrum is divided into two parts: a) *m/z* 200–700 and b) *m/z* 700–1,150).

The peak with the highest intensity corresponds to the anion AuCl_4_^−^, which was not identified in previous work. The cluster anions of the type Au_n_Cl_n+1_^-^ (discovered earlier), Au_n_Cl_n+3_^−^ and Au_n_Cl_n+5_^-^ show a significant intensity for n = 1–4. The anion Au_4_Cl_7_^−^ has the lowest intensity.

**FIGURE 2:**
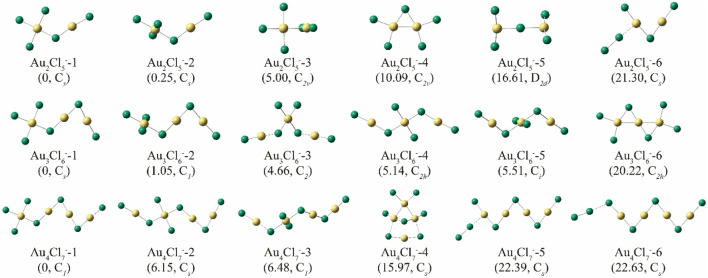
Structures of the top 6 isomers of Au_n_Cl_n+3_^-^ (n = 2–4). Their relative energies (ΔE, in kcal mol^-1^) and symmetry are shown in parentheses.

To determine the most stable structures of Au_n_Cl_n+3_^−^ (n = 2–4) cluster anions, extensive structures were generated, optimized and compared based on the cluster search program of NKCS (Zhou et al., 2021). Considering that the most stable structures of Au_n_Cl_n+1_^−^ are typically characterized by zigzag structures (Ma et al., 2019b), some similar structures were also generated by manually adding two Cl atoms. The top 6 isomers with the lowest energies of Au_n_Cl_n+3_^-^ (n = 2–4) are shown in Figure 2. For simplicity, we can divide the Au_2_Cl_5_ structures into two categories, those with Au-Au bonds and those without. The Au-Au bonds mentioned here refer to those with typical covalent bonding properties, with bond lengths of less than 2.72 Å (Cordero et al., 2008). And the aurophilic interactions, which typically have a distance between 2.72 and 3.32 Å, are not considered to be the Au-Au bonds discussed here (Sculfort and Braunstein, 2011).

**FIGURE 3:**
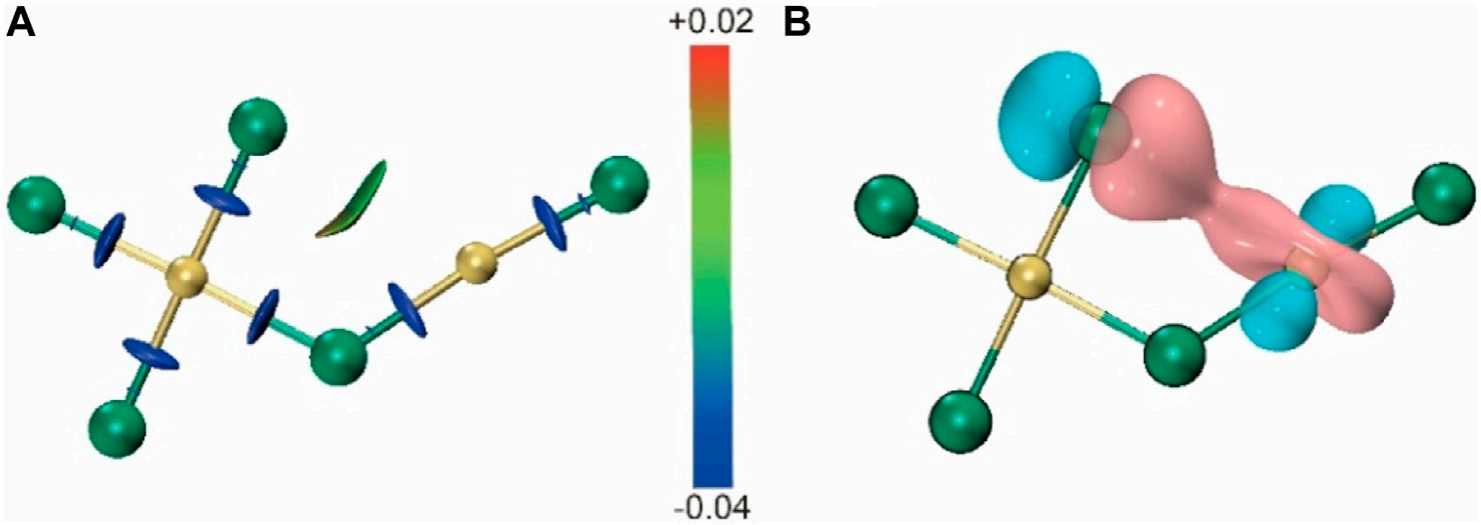
**(A)** IRI calculated for Au_2_Cl_5_^−^-1 cluster ions. **(B)** AdNDP calculated for Au_2_Cl_5_^−^-1 cluster ions.

For Au_2_Cl_5_^−^, it can be stated that the first two isomers, Au_2_Cl_5_-1 and −2, are characterized by their zigzag skeleton, which is similar to the previously reported structure of Au_2_Cl_3_^−^ (Ma et al., 2019b). The two isomers, which have only Au-Cl connections, are very close in energy. Both can be considered as structures formed by the fusion of structural units of AuCl_4_^−^ and AuCl_2_^−^ by eliminating a Cl atom from two different angles. The difference in their dihedral angles leads to an energy gap of 0.25 kcal/mol. Further analysis based on the interaction region indicator (IRI) and adaptive natural density partitioning (AdNAP) (Zubarev and Boldyrev, 2008; Lu and Chen, 2021) shows that the energy difference is due to the in-plane interaction between the separated chlorine and gold atoms on both sides caused by the coordination between the d orbitals of the gold atoms and the p orbitals of the chlorine atoms (Figure 3). In both structures, the distance between the two gold atoms is greater than 3.32 Å, indicating the absence of the aurophilic interaction. The third stable isomer, Au_2_Cl_5_-3, is characterized by an Au-Au bond with a length of 2.72 Å and has a relative energy 5.00 kcal/mol higher than Au_2_Cl_5_-1. The planar structure of Au_2_Cl_5_-4 has an Au-Au bond with a length of 2.72 Å and a C_*2v*_ symmetry, but is 10.09 kcal/mol less stable than the top isomer. The isomer Au_2_Cl_5_-6 also has a zigzag skeleton and can be regarded as a structure formed by attaching a chlorine molecule to the gold atom in the most stable isomer of Au_2_Cl_3_^−^.

For the anion Au_3_Cl_6_^−^, the first two isomers are characterized by similar skeletons to Au_2_Cl_5_^−^ −1 and −2, but the energy difference due to the weak coordination in the plane between the gold and chlorine atoms has increased to 1.05 kcal/mol, much greater than that between Au_2_Cl_5_^−^ −1 and −2. The isomers of Au_3_Cl_6_^−^ −3, −4 and −5 also have similar structures, which can be treated as if they were formed by inserting the AuCl_4_ unit into the chain of Au_2_Cl_3_^−^. Therefore, the three isomers have similar energies. The isomer Au_2_Cl_5_^−^-6, which has a relative energy of 20.22 kcal/mol, is characterized by its planar structure with a C_*2h*_ symmetry and the two consecutive Au-Au bonds.

Similar results are found in the structures of Au_4_Cl_7_^−^. As shown in Figure 2, the most stable isomer of Au_4_Cl_7_^−^ can be regarded as a natural extension of the structures of Au_2_Cl_5_^−^-1 and Au_3_Cl_6_^−^-1. The isomers with an AuCl_4_ unit (Au_4_Cl_7_^−^-2) inserted in the centre of the zigzag skeleton can increase the energy by 6.15 and 6.48 kcal/mol, respectively (this corresponding to the isomers of Au_4_Cl_7_^−^ −3 and −4). Some isomers with Au-Au bonds are also observed with a relative energy of almost 16 kcal/mol. And the isomers of Au_4_Cl_7_^−^ −5 and −6 can be considered as products formed by attaching the Cl_2_ molecule to the frameworks of Au_4_Cl_5_^−^ at different positions.

**FIGURE 4:**
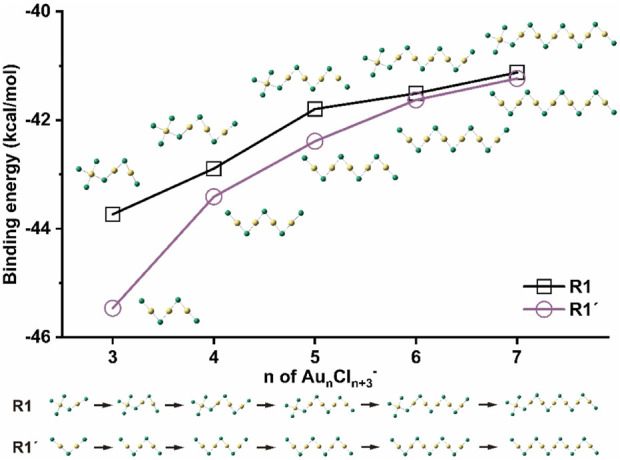
Binding energies of Au_n_Cl_n+3_^-^ (n = 3–7) on the pathway of R1 and those of Au_n_Cl_n+1_^-^ (n = 3–7) on the pathway of R1′.

The similarity and inheritance of the structures shown in Figure 2 also provide information about their probable growth processes. Roughly, these structures can be divided into two types: one is based on the zigzag skeleton of Au_n_Cl_n+1_^-^ clusters with two Cl atoms added at appropriate positions, and the other is the type with more complex topological connections that are not significantly related to the zigzag skeleton. Among them, the first category accounts for the majority. Considering the continuity of these structures, four different pathways have been proposed and investigated here. The structures of the most stable isomers of Au_n_Cl_n+3_^-^ (n = 2–4) are all characterized by a zigzag skeletons starting with the AuCl_4_^−^ unit. To better understand the corresponding growth process, cluster ions with similar structures but larger sizes (Au_n_Cl_n+3_^-^, n = 5–7) were also optimized, and their structures are shown in the proposed growth path R1 in Figure 4. This path R1 is very similar to the previously proposed path R1’ for Au_n_Cl_n+1_^-^ clusters (Ma et al., 2019b). The binding energy of each step in R1 is calculated based on:

$$Au_{n‐1}Cl_{{n+2}^{‐}}+AuCl\;\to \;Au_{n}Cl_{{n+3}^{‐}}\left(n=3‐7,withthestructuresshowninR1inFig.4\right)$$ (1)

For comparison, the binding energy of each step for clusters of Au_n_Cl_n+1_^-^ (n = 3–7) in R1’ is also calculated at the same level:

$${Au}_{n‐1}Cl_{n^{‐}}+AuCl\;\to \;{Au}_{n}Cl_{{n+1}^{‐}}\left(n=3‐7,withthestructuresshowninR1^{\prime }in\;Fig.\;4\right)$$ (2)

Figure 4 shows the binding energies of the two types of clusters as functions of the cluster sizes in the paths of R1 and R1’. The result shows that the growth process in R1 is exergonic, with ΔE between −41 and −44 kcal/mol, indicating that the pathway of R1 is thermodynamically favorable. As the size of the cluster increases and the zigzag skeleton chain becomes longer, the energy released decreases, which is very similar to the R1′-pathway in the case of Au_n_Cl_n+1_^−^. For clusters with a size of n > 6, the binding energies of both pathways are very close to each other and gradually converge to a stable value of about −41 kcal/mol.

Table 1 compares the most important geometric parameters of the structures in both paths. The Au-Au distance in Au_2_Cl_5_^−^ is 3.99 Å, which is significantly longer than that in Au_2_Cl_3_^−^ (3.46 Å). The electron localization function (ELF) profiles for both structures show no electron pair density between two Au atoms, indicating that there is no aurophilic interaction in either case. As the size of the cluster of Au_n_Cl_n+3_^-^ increases, the left-side terminal Au-Au distance, which represents the length between trivalent and monovalent gold atoms, gradually shortens to 3.82 Å. This distance is much longer than the terminal Au-Au distance in the case of Au_n_Cl_n+1_^-^ (2.97–2.93 Å) and also the sum of the van der Waals radii of two gold atoms (3.32 Å). However, all Au−Au distances between monovalent gold atoms in the clusters of Au_n_Cl_n+3_^-^ (n = 3–7) are 2.92 Å, which is slightly shorter than the distances in Au_n_Cl_n+1_^−^. These distances show the significant aurophilic interactions in the clusters, which are also confirmed by the ELF calculation results (Supplementary Figure S3). The results show that the structural difference between the clusters of Au_n_Cl_n+3_^-^ and Au_n_Cl_n+1_^-^ is mainly in the terminal trivalent gold unit. The structural features in the following zigzag skeletons, together with the binding energies, converge more and more to a fixed value with increasing size.

**TABLE 1:** Structural parameters (with the method of TPSSh and the basis sets of ECP60MDF for Au and aug-cc-pVTZ for Cl) of the isomers of Au_n_Cl_n+1_^-^ and Au_n_Cl_n+3_^-^ (n = 2–7) on the pathways of R1’ and R1 (ref to Supplementary Figure S4), respectively; Bond Lengths are in A and angles are in degrees.

Clusters	<Au^a^ Cl Au^b^	<Au^b^ Cl Au^b^	Au^a^−Au^b^	Au^b^−Au^b^
Au_2_Cl_3_^−^		95.94		3.46
Au_3_Cl_4_^−^		79.02		2.97
Au_4_Cl_5_^−^		79.43		2.96
Au_5_Cl_6_^−^		78.11		2.94
Au_6_Cl_7_^−^		77.91		2.94
Au_7_Cl_8_^−^				2.93
Au_2_Cl_5_^−^	117.14		3.99	
Au_3_Cl_6_^−^	112.95	77.29	3.91	2.92
Au_4_Cl_7_^−^	111.38	76.95	3.87	2.91
Au_5_Cl_8_^−^	109.83	77.43	3.84	2.92
Au_6_Cl_9_^−^	109.74	77.32	3.84	2.92
Au_7_Cl_10_^−^	109.21	77.47	3.82	2.92

a Representing Au(III),b representing Au(I).

**FIGURE 5:**
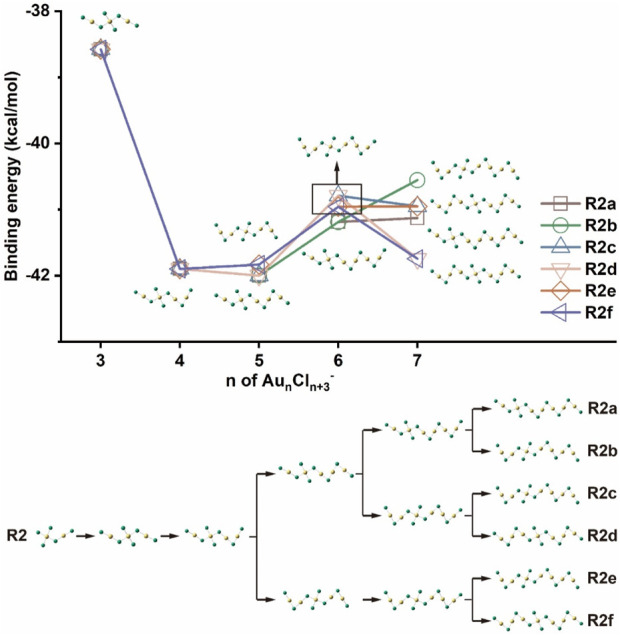
Binding energies of Au_n_Cl_n+3_^-^ (n = 3–7) according to the growth route of R2.

As already mentioned, Figure 2 shows another class of isomers that differ from those in R1 by a change in the position of the trivalent gold atom. Instead of the terminal position, the trivalent gold atom can also be located anywhere within the zigzag chain, leading to various isomers with total energies 5–7 kcal/mol higher than the corresponding isomers with the lowest energies. The pathways for the formation of such isomers are discussed and shown in Figure 5 as route 2 (R2). In pathway R1, the trivalent gold atoms are fixed at one end of the chain, and the clusters extend only along the side of the monovalent gold, while in the first step of R2, the chain extends on the other side of the Au(III) unit and forms a sandwich-like Au(III) inside. The chain can then be further extended on both sides of the Au(I) unit to form different or identical structures along different paths. As can be seen in Figure 5, the growth path away from the Au(III) unit releases more energy (R2a), which is thermodynamically advantageous. The result is consistent with that reported in R1, i.e., the Au(I) units tend to bond with each other to strengthen the aurophilic interactions so that they stay away from the Au(III) unit. Therefore, the binding energy in the most favorable pathway in R2 (R2a) also tends to stabilize at −41 kcal/mol as the cluster size increases, which is similar to that in the R1 pathway. As shown in Supplementary Table S1, aurophilic interactions can be clearly found when the distance between the nearby Au atoms is taken into account. Here, no aurophilic interaction can be found between the Au(III) and Au(I) atoms, but the distances between the nearby Au(I) atoms in the isomers shown in R2 are typically 2.87–2.90 Å, even shorter than those of the most stable isomers reported in R1.

**FIGURE 6:**
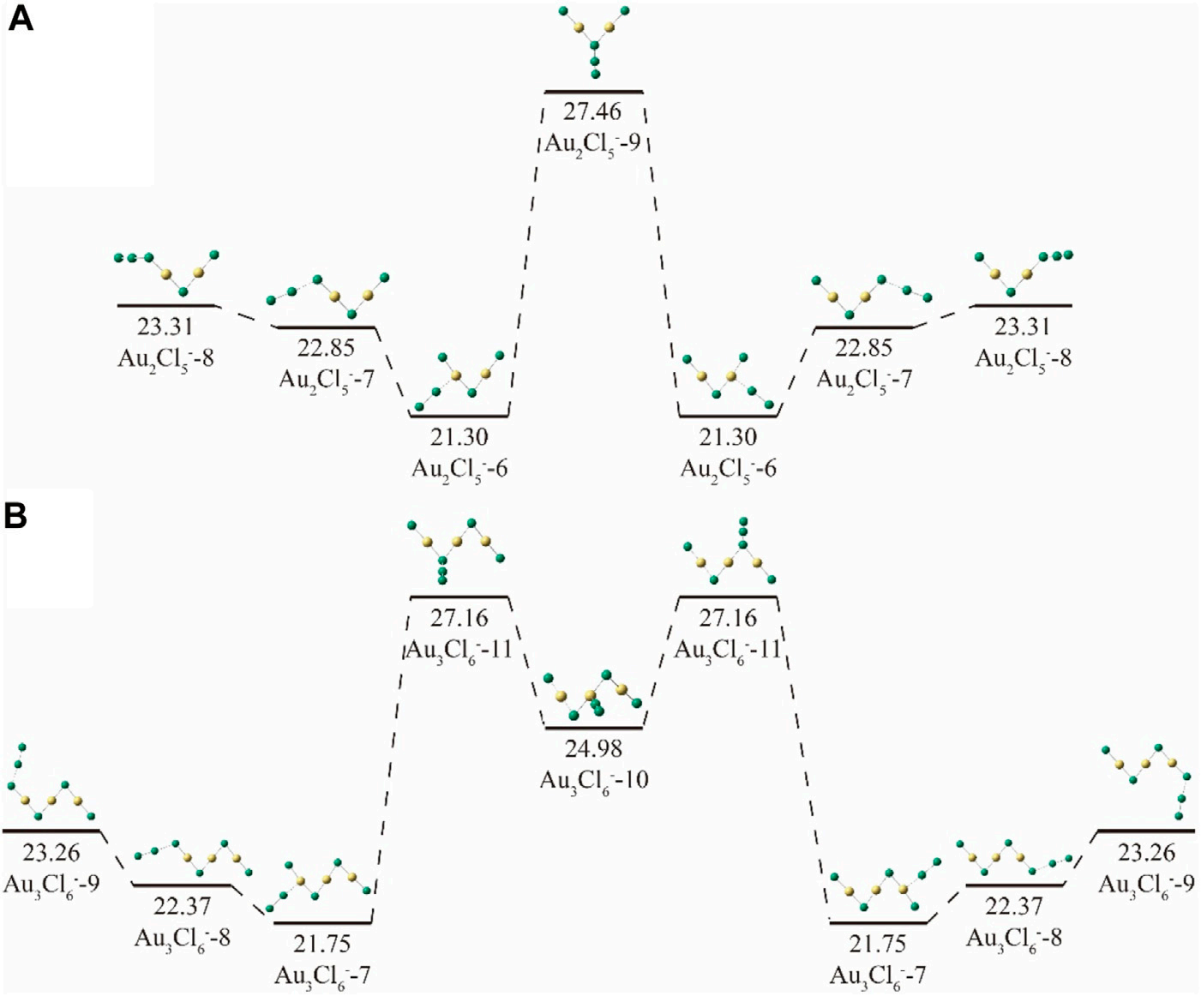
The relative energies of **(A)** Au_2_Cl_5_^−^-n (n = 6–9) and **(B)** Au_n_Cl_n+3_^-^ (n = 7–11) cluster ions.

All isomers in R1 and R2 contain an Au(III) unit. However, it is possible that the cluster can also form by appending a Cl_2_ unit to the previously reported zigzag skeleton of Au_n_Cl_n+1_^-^, as shown by the isomers of Au_2_Cl_5_^−^-6, Au_3_Cl_6_^−^-7 and Au_4_Cl_7_^−^-5 shown in Figure 2 and Supplementary Figure S2. These isomers are interesting due to their structural diversity and possible isomerization pathways, even though their relative energies are higher than those of the most stable isomers by more than 20 kcal/mol. They can be treated as complexes formed by the anchoring of a Cl_2_ unit to the skeleton of Au_n_Cl_n+1_^−^. It has been shown that each atom of the zigzag skeleton can act as an anchor. Even with the same anchoring position, different isomers can be formed due to the different anchoring angles. Figure 6A shows these four isomers of Au_2_Cl_5_^−^. Among these isomers, the one in which the Cl_2_ unit is anchored to the Au(I) atom (Au_2_Cl_5_^−^-6) has the lowest energy, while the isomer in which the Cl_2_ unit is anchored to the middle Cl atom has a higher relative energy by 6.16 kcal/mol. Among the isomers anchored at the edge of the Cl atom, two isomers are both stable due to their anchoring directions, with energies of 2.01 and 1.55 kcal/mol higher than the isomer of Au_2_Cl_5_^−^-6. Although the isomerization barriers between these isomers have not yet been calculated, it is believed that it is possible to move the Cl_2_ unit along the zigzag edge of the skeleton of Au_n_Cl_n+1_^-^ by manipulating the Cl_2_ unit by appropriate experimental methods. In addition to these isomers shown here, there are other isomers in which the two Cl atoms (instead of the Cl_2_ unit) are anchored to the edge of the skeleton from both sides (Au_2_Cl_5_^−^-10 and Au_2_Cl_5_^−^-11, see Supplementary Figure S1) or from both Cl atoms (Au_2_Cl_5_^−^-12, see Supplementary Figure S1), although their energies are much higher. For Au_3_Cl_6_^−^, more anchor positions were found for the Cl_2_ unit (Figure 6B). Similar to Au_2_Cl_5_^−^, the isomer anchored with the edge Au atom is the most stable isomer of this type, and the isomer anchored with the edge Cl atom is the second most stable. Other isomers anchored with middle Cl atoms or Au atoms in the zigzag skeleton are less stable than the most stable isomer by 5.41 and 2.18 kcal/mol, respectively.

**FIGURE 7:**
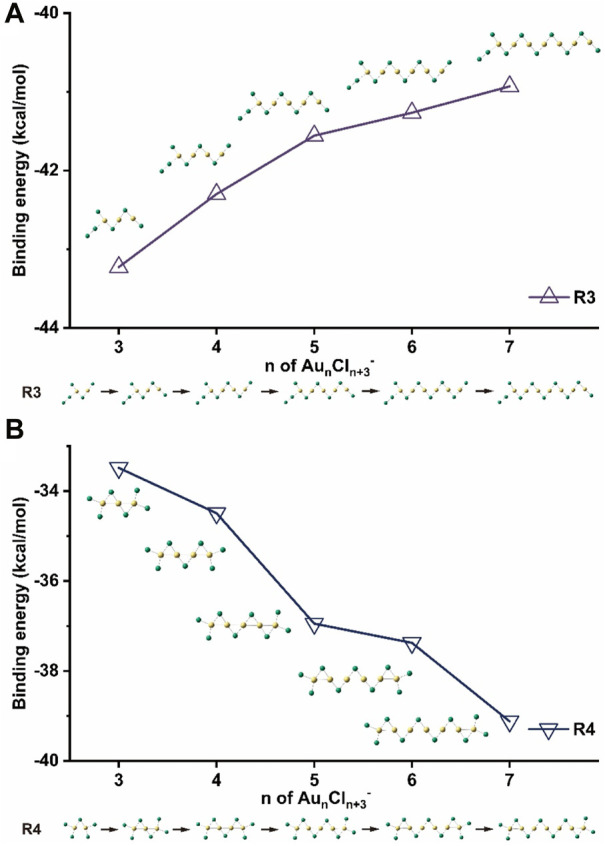
Binding energies of Au_n_Cl_n+3_^-^ (n = 3–7) according to growth routes of **(A)** R3, and **(B)** R4.

Starting from the most stable structure, in which the Cl_2_ unit is anchored to the Au atom at the edge, a different pathway for the growth of isomers of this type is proposed and shown in Figure 7 as route 3 (R3). The binding energies and the main geometrical parameters of the structures in R3 are shown and listed in Figure 7 and Supplementary Table S2, respectively. Similar to the paths R1’ for Au_n_Cl_n+1_^-^ and R1 for Au_n_Cl_n+3_^-^, the absolute values of the binding energy decrease from 43 kcal/mol to 41 kcal/mol with increasing size of the cluster. Obviously, the aurophilic interaction is filled with the zigzag chains, with a typical Au-Au distance of 2.94 Å and Au-Cl-Au angle of 78^°^, both of which are close to those in the clusters of Au_n_Cl_n+1_^−^.

**FIGURE 8:**
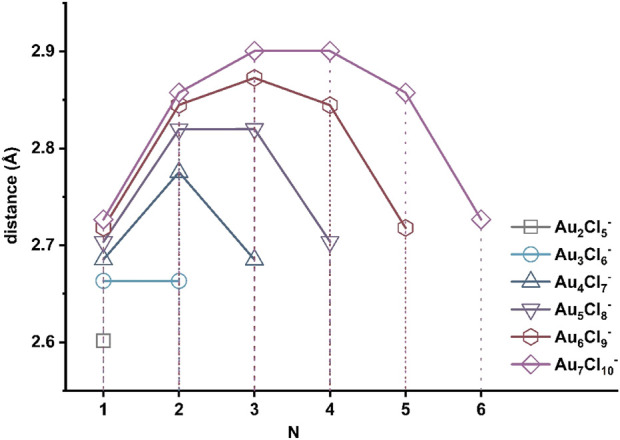
Adjacent Au-Au(N) lengths (in A) in Au_n_Cl_n+3_^-^ (n = 2–7) isomers, which are shown in the pathway of R4 (Figure 7). The N in the abscissa indicates the *N*th Au-Au distance counted from left terminals to right terminals.

The isomers of Au_2_Cl_5_^−^-4 and Au_3_Cl_6_^−^-5 with symmetric planar structure are different from the zigzag structures. Although their relative energies are higher, the Au-Au bonds present inside the clusters suggest that they may have a completely different growth path. Therefore, the corresponding isomers with n = two to seven and the path of R4 were calculated and shown in Figure 7. The heat released during these exothermic processes increases with the size of the cluster. Figure 8 shows the distances between nearby gold atoms in these structures, where the N stands for the *N*th gold atom in the corresponding structures counted from the left. As can be seen in the figure, the Au-Au distances in the four clusters increase with n ≥ 4 along the molecular chains, reaching maximum values and then decreasing to values close to the initial values. The Au-Au distances in clusters with n ≤ 3 are always smaller than 2.72 Å, highlighting the dominant role of Au-Au bonding, while the distances in the middle parts of clusters with larger sizes (n = 6, 7) are between 2.72 Å and 3.32 Å, indicating the existence of an aurophilic interaction (Supplementary Table S3). The structural transition from Au-Au bonding to aurophilic interaction occurs for the cluster with n = 4. It can be naturally assumed that aurophilic interactions along the chains are a feature of isomers with larger sizes in the R4 pathway, although the Au-Au bonds may still exist at the two ends.

It should be mentioned that the cluster of Au_n_Cl_n+5_^-^ (n = 2, 3, 4) were also identified in the LDI mass spectrum (Figure 1). The preliminary theoretical calculations for these culsters show that their structures may retain the similar configuration. Due to the diversity and complexity of their structures, further research is still underway on their most stable structures and growth pathways. We hope these results can help to understand the structure, interactions, and properties of such cluster ions more systematically.

## 4 Conclusion

In summary, cluster anions of Au_n_Cl_n+3_^-^ (n = 2–4) were obtained by LDI mass spectrometry. Their most stable isomers were studied in detail and finally optimized at the TPSSh/aug-cc-pVTZ/ECP60MDF level. The results show that the isomers with the lowest energy for the clusters Au_n_Cl_n+3_^-^ (n = 2–4) have similar structures, all characterized by a zigzag skeleton. Compared to the most stable structures of Au_n_Cl_n+1_^-^ reported previously, the two Cl atoms have been added to the edge gold atom to give it an oxidation state of +3, canceling the original aurophilic interaction with the edge gold atom (if present). Furthermore, the gold-chlorine coordination results in the most stable isomers having a planar structure. Based on these results, four growth pathways for Au_n_Cl_n+3_^-^ (n = 2–7) clusters are proposed. These pathways are exothermic processes, and the Au(I)···Au(I) interactions are present in all of them. For the pathway R1, which connects all the most stable isomers of the corresponding clusters, it is found that the growth of the clusters tends to occur at the end of the Au(I) due to the aurophilic interactions between Au(I) and Au(I), and the Au(III) preferentially remains at the other end of the zigzag chain. For the pathways of R1, R2 and R3, the binding energies tend to stabilize at −41 kcal/mol as the size of the cluster increases.

The pathway of the R4 is different. In this pathway, which contains an Au-Au bond in its initial structures (n ≤ 3), the released heat increases and the distance between the intermediate gold atoms increases with increasing cluster size, which eventually leads to the transformation of the intermediate Au-Au bond into an aurophilic interaction.

All these pathways lead to structures with similar one-dimensional zigzag chains stabilized by aurophilic interactions with similar energies. These results not only help us to better understand the size effect on the structure and aurophilic interactions of these clusters, but may also reflect some new information about the active but elusive species in related catalytic reactions or nanoparticle synthesis processes.

## Data Availability

The datasets presented in this study can be found in online repositories. The names of the repository/repositories and accession number(s) can be found in the article/Supplementary Material.

## References

[B1] Barngrover B. M. Aikens C. M. (2012). The golden pathway to thiolate-stabilized nanoparticles: following the formation of gold(I) thiolate from gold(III) chloride. J. Am. Chem. Soc. 134, 12590–12595. 10.1021/ja303050s 22827488

[B2] Barngrover B. M. Manges T. J. Aikens C. M. (2015). Prediction of nonradical Au(0)-containing precursors in nanoparticle growth processes. J. Phys. Chem. A 119, 889–895. 10.1021/jp509676a 25580885

[B3] Brust M. Walker M. Bethell D. Schiffrin D. J. Whyman R. (1994). Synthesis of thiol-derivatised gold nanoparticles in a two-phase Liquid-Liquid system. J. Chem. Soc. Chem. Commun. 7, 801–802. 10.1039/c39940000801

[B4] Cao S. Yang M. Elnabawy A. O. Trimpalis A. Li S. Wang C. (2019). Single-atom gold oxo-clusters prepared in alkaline solutions catalyse the heterogeneous methanol self-coupling reactions. Nat. Chem. 11, 1098–1105. 10.1038/s41557-019-0345-3 31636391

[B5] Cordero B. Gómez V. Platero-Prats A. E. Revés M. Echeverría J. Cremades E. (2008). Covalent radii revisited. Dalton Trans., 2832–2838. 10.1039/b801115j 18478144

[B6] Davies C. J. Miedziak P. J. Brett G. L. Hutchings G. J. (2016). Vinyl chloride monomer production catalysed by gold: a review. Chin. J. Catal. 37, 1600–1607. 10.1016/s1872-2067(16)62482-8

[B7] Goulet P. J. G. Lennox R. B. (2010). New insights into brust-schiffrin metal nanoparticle synthesis. J. Am. Chem. Soc. 132, 9582–9584. 10.1021/ja104011b 20568767

[B8] Jerabek P. von der Esch B. Schmidbaur H. Schwerdtfeger P. (2017). Influence of relativistic effects on bonding modes in M(II) dinuclear complexes (M = Au, Ag, and Cu). Inorg. Chem. 56, 14624–14631. 10.1021/acs.inorgchem.7b02434 29135228

[B9] Lemke K. H. (2014). Gold chloride clusters with Au(III) and Au(I) probed by FT-ICR mass spectrometry and MP2 theory. Phys. Chem. Chem. Phys. 16, 7813. 10.1039/c3cp55109a 24643288

[B10] Li Y. Zaluzhna O. Xu B. Gao Y. Modest J. M. Tong Y. J. (2011). Mechanistic insights into the brust-schiffrin two-phase synthesis of organo-chalcogenate-protected metal nanoparticles. J. Am. Chem. Soc. 133, 2092–2095. 10.1021/ja1105078 21268580

[B11] Lu T. Chen F. (2011). Meaning and functional form of the electron localization function. Acta Phys. Chim. Sin. 27, 2786–2792. 10.3866/pku.Whxb20112786

[B12] Lu T. Chen F. (2012). Multiwfn: a multifunctional wavefunction analyzer. J. Comput. Chem. 33, 580–592. 10.1002/jcc.22885 22162017

[B13] Lu T. Chen Q. (2021). Interaction region indicator: a simple real space function clearly revealing both chemical bonds and weak interactions. Chemistry–Methods 1, 231–239. 10.1002/cmtd.202100007

[B14] Ma Y. Bian S. Shi Y. Fan X. Kong X. (2019a). Greatly enhanced electron affinities of Au_2n_Cl clusters (n = 1–4): effects of chlorine doping. ACS Omega 4, 17295–17300. 10.1021/acsomega.9b01981 31656903 PMC6811865

[B15] Ma Y. Bian S. Shi Y. Fan X. Kong X. (2019b). Size effect on aurophilic interaction in gold-chloride cluster anions of Au_n_Cl_n+1_ ^-^ (2 ≤ n ≤ 7). ACS Omega 4, 650–654. 10.1021/acsomega.8b02907 31459354 PMC6649055

[B16] Murray R. W. (2008). Nanoelectrochemistry: metal nanoparticles, nanoelectrodes, and nanopores. Chem. Rev. 108, 2688–2720. 10.1021/cr068077e 18558753

[B17] Rabilloud F. (2012). Structure and bonding in coinage metal halide clusters M_n_X_n_, M = Cu, Ag, Au; X = Br, I; n = 1–6. J. Phys. Chem. A 116, 3474–3480. 10.1021/jp300756h 22432857

[B18] Sardar R. Funston A. M. Mulvaney P. Murray R. W. (2009). Gold nanoparticles: past, present, and future. Langmuir 25, 13840–13851. 10.1021/la9019475 19572538

[B19] Scherbaum F. Grohmann A. Huber B. Krüger C. Schmidbaur H. (1988). Aurophilicity as a consequence of relativistic effects: the hexakis(triphenylphosphaneaurio)methane dication [(Ph_3_PAu)_6_C]^2⊕^ . Angew. Chem. Int. Ed. 27, 1544–1546. 10.1002/anie.198815441

[B20] Schmidbaur H. Graf W. Müller G. (1988a). Weak intramolecular bonding relationships: the conformation-determining attractive interaction between gold(I) centers. Angew. Chem. Int. Ed. 27, 417–419. 10.1002/anie.198804171

[B21] Schmidbaur H. Scherbaum F. Huber B. Müller G. (1988b). Polyauriomethane compounds. Angew. Chem. Int. Ed. 27, 419–421. 10.1002/anie.198804191

[B22] Schwerdtfeger P. Krawczyk R. P. Hammerl A. Brown R. (2004). A comparison of structure and stability between the group 11 halide tetramers M_4_X_4_ (M = Cu, Ag, or Au; X = F, Cl, Br, or I) and the group 11 chloride and bromide phosphanes (XMPH_3_)_4_ . Inorg. Chem. 43, 6707–6716. 10.1021/ic0492744 15476370

[B23] Sculfort S. Braunstein P. (2011). Intramolecular d^10^-d^10^ interactions in heterometallic clusters of the transition metals. Chem. Soc. Rev. 40, 2741–2760. 10.1039/c0cs00102c 21331417

[B24] Srivastava A. K. Misra N. (2014). The highest oxidation state of Au revealed by interactions with successive Cl ligands and superhalogen properties of AuCl_n_ (n = 1-6) species. Int. J. Quantum Chem. 114, 1513–1517. 10.1002/qua.24717

[B25] Templeton A. C. Wuelfing W. P. Murray R. W. (2000). Monolayer-protected cluster molecules. Acc. Chem. Res. 33, 27–36. 10.1021/ar9602664 10639073

[B26] Theilacker K. Schlegel H. B. Kaupp M. Schwerdtfeger P. (2015). Relativistic and solvation effects on the stability of gold(III) halides in aqueous solution. Inorg. Chem. 54, 9869–9875. 10.1021/acs.inorgchem.5b01632 26421633

[B27] Zhang J. Simon M. Golz C. Alcarazo M. (2020). Gold‐catalyzed atroposelective synthesis of 1,1′‐Binaphthalene‐2,3′‐diols. Angew. Chem. Int. Ed. 59, 5647–5650. 10.1002/anie.201915456 PMC7155015 31859408

[B28] Zhou M. Xu Y. Cui Y. Zhang X. Kong X. (2021). Search for global minimum structures of P_2n+1_ ^+^ (n = 1–15) using xTB-based basin-hopping algorithm. Front. Chem. 9, 694156. 10.3389/fchem.2021.694156 34381759 PMC8350033

[B29] Zubarev D. Y. Boldyrev A. I. (2008). Developing paradigms of chemical bonding: adaptive natural density partitioning. Phys. Chem. Chem. Phys. 10, 5207. 10.1039/b804083d 18728862

