# Supplementary material for: Structures and growth pathways of AunCln+3 - (n ≤ 7) cluster anions
Source: Front Chem. 2024 Mar 11;12:1382443. doi: 10.3389/fchem.2024.1382443 (PMC11027128; doi:10.3389/fchem.2024.1382443)
Supplement: Supplementary file 1 [file DataSheet1.docx]

**SUPPORTING INFORMATION**

Structures and Growth Pathways of Au_n_Cl_n+3_^-^ (n ≤ 7) Cluster Anions

Shiyin Xu^1^, Xinhe Liu^1^, Yameng Hou^1^, Min Kou^1^, Xinshi Xu^1^,Filip Veljković^2^, Suzana Veličković^2*^, Xianglei Kong^1,3*^

*^1^ State Key Laboratory of Elemento-Organic Chemistry, Frontiers Science Center for New Organic Matter, College of Chemistry, Nankai University, Tianjin 300071, China.*

*^2^ ‘‘VINCA” Institute of Nuclear Sciences - National Institute of the Republic of Serbia, University of Belgrade, Mike Petrovica Alasa 12-14, 11351 Belgrade, Serbia*

*^3^ Tianjin Key Laboratory of Biosensing and Molecular Recognition, College of Chemistry, Nankai University, Tianjin 300071, China*

**Figure S1** Structures of the top 12 isomers of Au_2_Cl_5_^-^ cluster ions. Their relative energies (ΔE, in kcal mol^-1^) and symmetry are shown in parentheses.

**Figure S2** Structures of the top 12 isomers of Au_3_Cl_6_^-^ cluster ions. Their relative energies (ΔE, in kcal mol^-1^) and symmetry are shown in parentheses.

**Figure S3** ELFs calculated for the most stable isomers of Au_n_Cl_n+3_^-^ and Au_n_Cl_n+1_^-^ (n = 2-4) cluster ions.

**Table S1** TPSSh structural parameters (with the sets of ECP60MDF for Au and aug-cc-pVTZ for Cl) of Au_n_Cl_n+3_^-^ (n = 2-7) on R2. Bond lengths are in Å’s and angles are in degrees.

**Table S2** TPSSh structural parameters (with the sets of ECP60MDF for Au and aug-cc-pVTZ for Cl) of Au_n_Cl_n+3_^-^ (n = 2-7) on R3. Bond lengths are in Å’s and angles are in degrees.

**Table S3** TPSSh structural parameters (with the sets of ECP60MDF for Au and aug-cc-pVTZ for Cl) of Au_n_Cl_n+3_^-^ (n = 2-7) on R4. Bond lengths are in Å’s. The N represents the N^th^ gold atom started from the left end in corresponding structures.

**Cartesian coordinate**s (in Å) for a) Au_2_Cl_5_^-^-1 b) Au_3_Cl_6_^-^-1 and c) Au_4_Cl_7_^-^-1 clusters at the level of TPSSh.

**Figure S1.** Structures of the top 12 isomers of Au_2_Cl_5_^-^ cluster ions. Their relative energies (ΔE, in kcal mol^-1^) and symmetry are shown in parentheses.


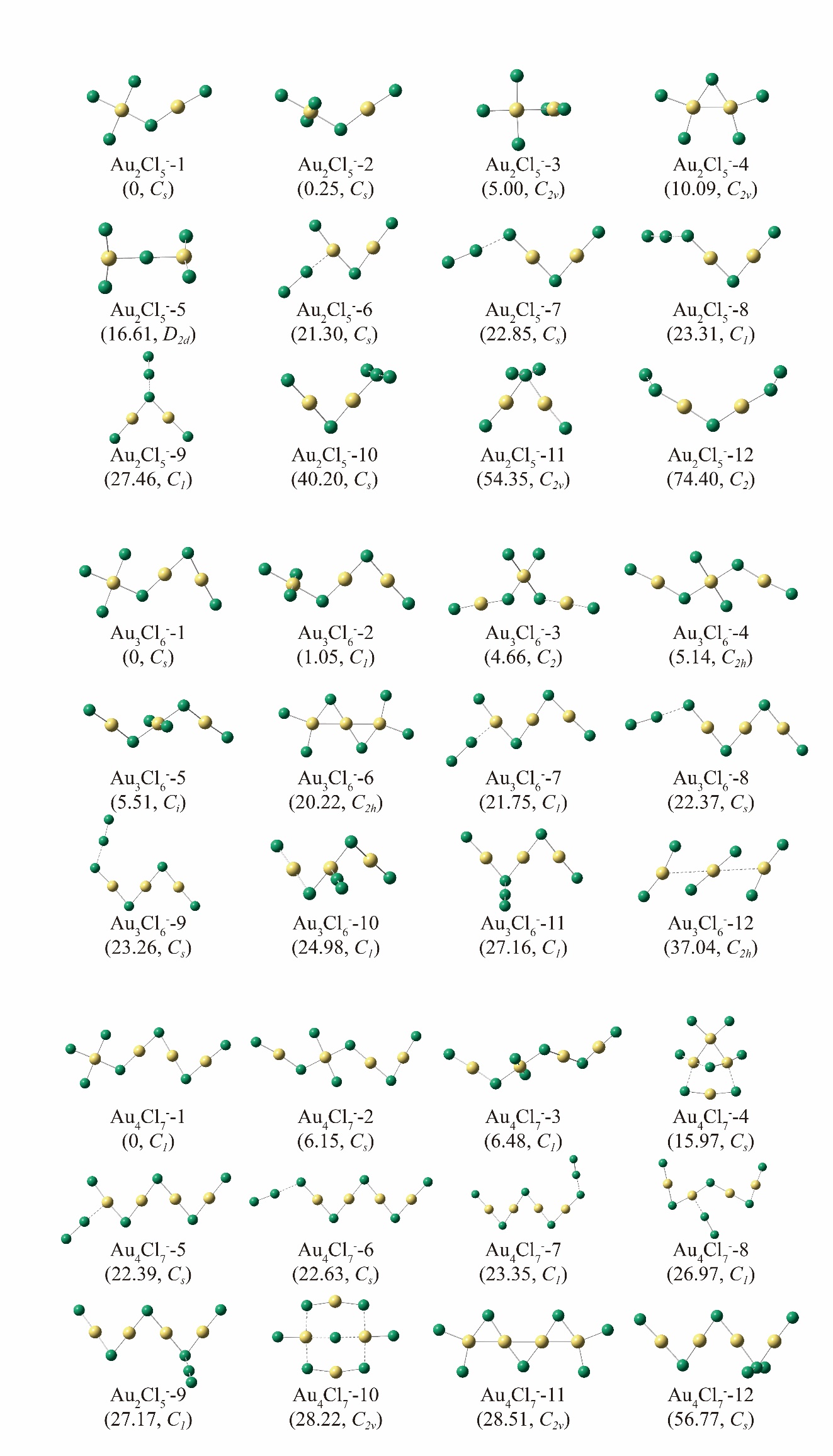


**Figure S2.** Structures of the top 12 isomers of Au_3_Cl_6_^-^ cluster ions. Their relative energies (ΔE, in kcal mol^-1^) and symmetry are shown in parentheses.


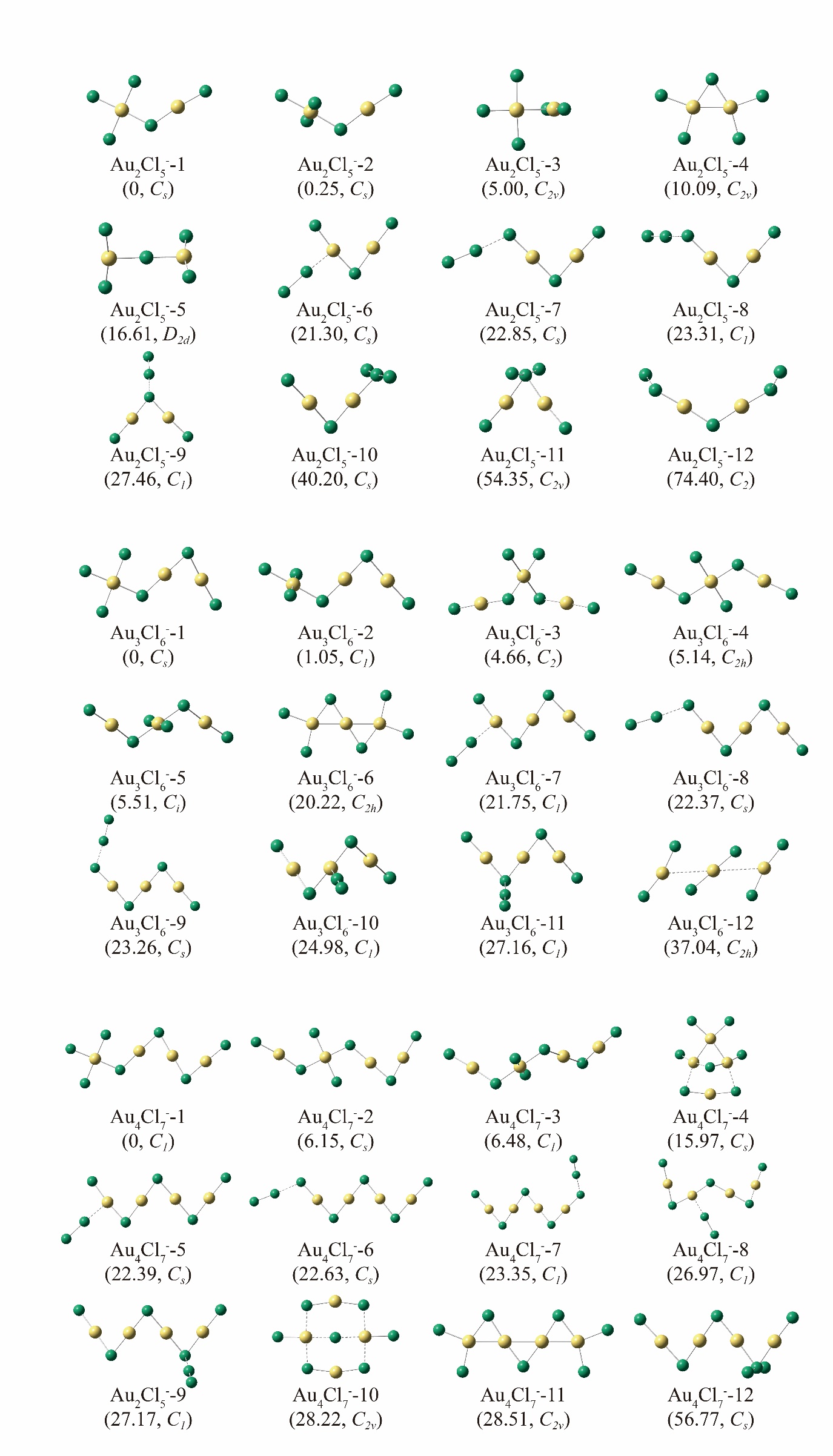


**Figure S3.** ELFs calculated for the most stable isomers of Au_n_Cl_n+3_^-^ and Au_n_Cl_n+1_^-^ (n = 2-4) cluster ions.


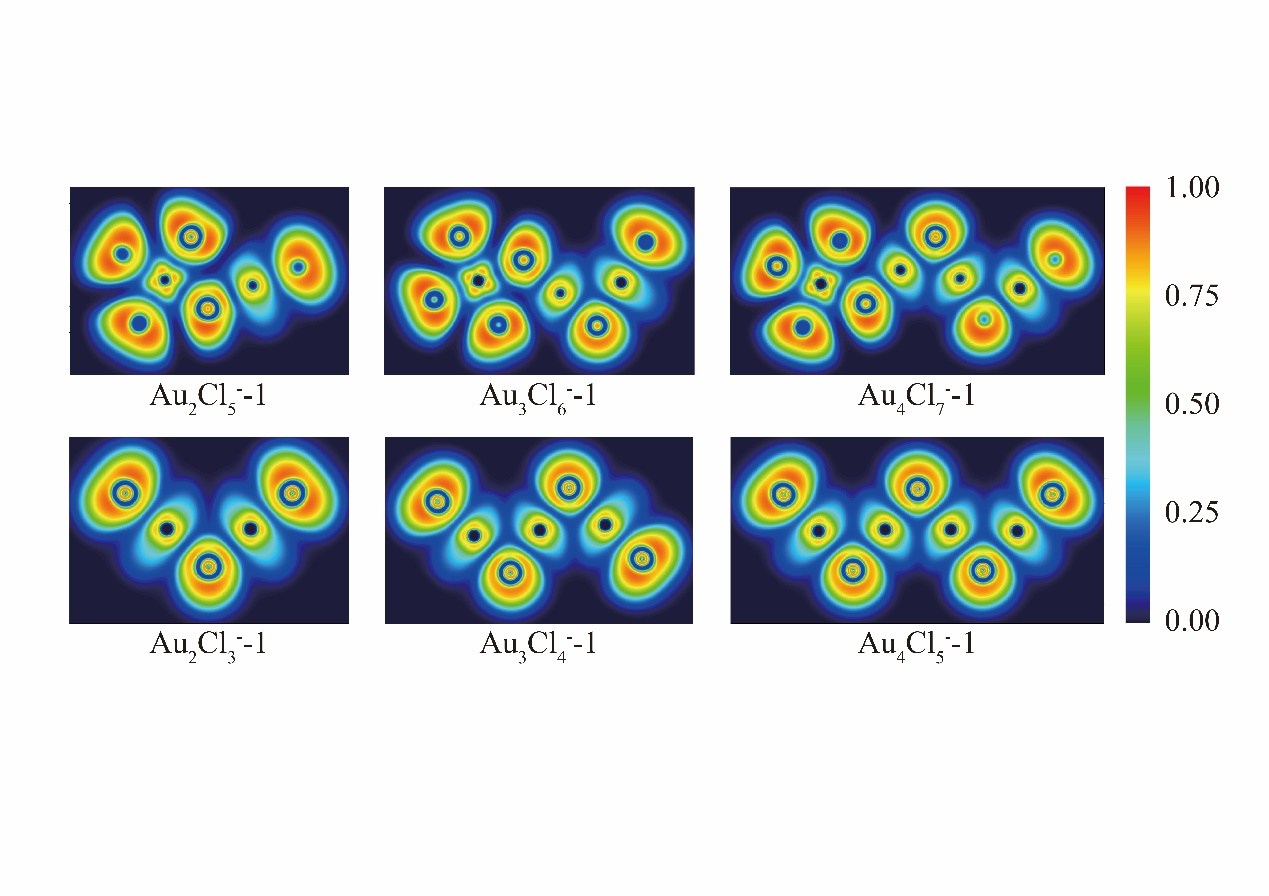


**Table S1.** TPSSh structural parameters (with the sets of ECP60MDF for Au and aug-cc-pVTZ for Cl) of Au_n_Cl_n+3_^-^ (n = 2-7) on R2. Bond lengths are in Å’s and angles are in degrees.

| R2a | <Au Cl Au^a^ | <Au Cl Au^b^ | Au−Au^a^ | Au−Au^b^ |
| --- | --- | --- | --- | --- |
| Au_2_Cl_5_^-^ | 117.14 |  | 3.99 |  |
| Au_3_Cl_6_^-^ | 116.60 |  | 3.97 |  |
| Au_4_Cl_7_^-^ | 115.57 | 76.21 | 3.94 | 2.89 |
| Au_5_Cl_8_^-^ | 114.90 | 76.23 | 3.93 | 2.89 |
| Au_6_Cl_9_^-^ | 114.50 | 76.52 | 3.92 | 2.90 |
| Au_7_Cl_10_^-^ | 113.80 | 76.70 | 3.90 | 2.90 |

| R2b | <Au Cl Au^a^ | <Au Cl Au^b^ | Au−Au^a^ | Au−Au^b^ |
| --- | --- | --- | --- | --- |
| Au_2_Cl_5_^-^ | 117.14 |  | 3.99 |  |
| Au_3_Cl_6_^-^ | 116.60 |  | 3.97 |  |
| Au_4_Cl_7_^-^ | 115.57 | 76.21 | 3.94 | 2.89 |
| Au_5_Cl_8_^-^ | 114.90 | 76.23 | 3.93 | 2.89 |
| Au_6_Cl_9_^-^ | 114.50 | 76.52 | 3.92 | 2.90 |
| Au_7_Cl_10_^-^ | 112.93 | 75.83 | 3.89 | 2.88 |

| R2c | <Au Cl Au^a^ | <Au Cl Au^b^ | Au−Au^a^ | Au−Au^b^ |
| --- | --- | --- | --- | --- |
| Au_2_Cl_5_^-^ | 117.14 |  | 3.99 |  |
| Au_3_Cl_6_^-^ | 116.60 |  | 3.97 |  |
| Au_4_Cl_7_^-^ | 115.57 | 76.21 | 3.94 | 2.89 |
| Au_5_Cl_8_^-^ | 114.90 | 76.23 | 3.93 | 2.89 |
| Au_6_Cl_9_^-^ | 113.19 | 75.52 | 3.89 | 2.87 |
| Au_7_Cl_10_^-^ | 112.93 | 75.83 | 3.89 | 2.88 |

| R2d | <Au Cl Au^a^ | <Au Cl Au^b^ | Au−Au^a^ | Au−Au^b^ |
| --- | --- | --- | --- | --- |
| Au_2_Cl_5_^-^ | 117.14 |  | 3.99 |  |
| Au_3_Cl_6_^-^ | 116.60 |  | 3.97 |  |
| Au_4_Cl_7_^-^ | 115.57 | 76.21 | 3.94 | 2.89 |
| Au_5_Cl_8_^-^ | 114.90 | 76.23 | 3.93 | 2.89 |
| Au_6_Cl_9_^-^ | 113.19 | 75.52 | 3.89 | 2.87 |
| Au_7_Cl_10_^-^ | 112.78 | 75.42 | 3.89 | 2.86 |

| R2e | <Au Cl Au^a^ | <Au Cl Au^b^ | Au−Au^a^ | Au−Au^b^ |
| --- | --- | --- | --- | --- |
| Au_2_Cl_5_^-^ | 117.14 |  | 3.99 |  |
| Au_3_Cl_6_^-^ | 116.60 |  | 3.97 |  |
| Au_4_Cl_7_^-^ | 115.57 | 76.21 | 3.94 | 2.89 |
| Au_5_Cl_8_^-^ | 113.80 | 75.43 | 3.91 | 2.87 |
| Au_6_Cl_9_^-^ | 113.19 | 75.52 | 3.89 | 2.87 |
| Au_7_Cl_10_^-^ | 112.93 | 75.83 | 3.89 | 2.88 |

| R2f | <Au Cl Au^a^ | <Au Cl Au^b^ | Au−Au^a^ | Au−Au^b^ |
| --- | --- | --- | --- | --- |
| Au_2_Cl_5_^-^ | 117.14 |  | 3.99 |  |
| Au_3_Cl_6_^-^ | 116.60 |  | 3.97 |  |
| Au_4_Cl_7_^-^ | 115.57 | 76.21 | 3.94 | 2.89 |
| Au_5_Cl_8_^-^ | 113.80 | 75.43 | 3.91 | 2.87 |
| Au_6_Cl_9_^-^ | 113.19 | 75.52 | 3.89 | 2.87 |
| Au_7_Cl_10_^-^ | 112.78 | 75.42 | 3.89 | 2.86 |

**Table S2.** TPSSh structural parameters (with the sets of ECP60MDF for Au and aug-cc-pVTZ for Cl) of Au_n_Cl_n+3_^-^ (n = 2-7) on R3. Bond lengths are in Å’s and angles are in degrees.

| R3 | <Au Cl Au^a^ | <Au Cl Au^b^ | Au−Au^a^ | Au−Au^b^ |
| --- | --- | --- | --- | --- |
| Au_2_Cl_5_^-^ |  | 78.21 |  | 2.95 |
| Au_3_Cl_6_^-^ |  | 77.99 |  | 2.94 |
| Au_4_Cl_7_^-^ |  | 77.65 |  | 2.93 |
| Au_5_Cl_8_^-^ |  | 78.17 |  | 2.94 |
| Au_6_Cl_9_^-^ |  | 77.96 |  | 2.94 |
| Au_7_Cl_10_^-^ |  | 78.03 |  | 2.94 |

**Table S3.** TPSSh structural parameters (with the sets of ECP60MDF for Au and aug-cc-pVTZ for Cl) of Au_n_Cl_n+3_^-^ (n = 2-7) on R4. Bond lengths are in Å’s. The N represents the N^th^ gold atom started from the left end in corresponding structures.

| R4 | N = 1 | N = 2 | N = 3 | N = 4 | N = 5 | N = 6 |
| --- | --- | --- | --- | --- | --- | --- |
| Au_2_Cl_5_^-^ | 2.60 |  |  |  |  |  |
| Au_3_Cl_6_^-^ | 2.66 | 2.66 |  |  |  |  |
| Au_4_Cl_7_^-^ | 2.69 | 2.78 | 2.69 |  |  |  |
| Au_5_Cl_8_^-^ | 2.70 | 2.82 | 2.82 | 2.70 |  |  |
| Au_6_Cl_9_^-^ | 2.72 | 2.84 | 2.87 | 2.84 | 2.72 |  |
| Au_7_Cl_10_^-^ | 2.73 | 2.86 | 2.90 | 2.90 | 2.86 | 2.73 |

**Cartesian coordinates** (in Å) for a) Au_2_Cl_5_^-^-1 b) Au_3_Cl_6_^-^-1 and c) Au_4_Cl_7_^-^-1 clusters at the level of TPSSh.

a) Au_2_Cl_5_^-^

Cl 0.22781400 -1.2552350 0.32297100

Cl -2.8892990 -1.9365820 -0.1922640

Cl -0.5362490 2.00668400 0.26157700

Au 2.26394000 -0.1976670 0.00533900

Au -1.7233200 0.04872000 0.02588900

Cl 4.34733400 0.63812600 -0.2876700

Cl -3.6618950 1.23917300 -0.2497320

b) Au_3_Cl_6_^-^-1

Au -3.1472510 0.16130800 0.04934900

Cl 4.75061300 1.99811400 0.37922600

Cl -1.0128660 1.14371000 -0.2201240

Au 0.70882400 -0.4122390 -0.1986430

Au 3.57854100 0.07959900 0.07798900

Cl 2.45379900 -1.9608820 -0.2287830

Cl -2.2042170 -1.9226920 0.32972300

Cl -4.04958000 2.26382300 -0.2392380

Cl -5.23592400 -0.72588300 0.31055300

c) Au_4_Cl_7_^-^-1

Cl 3.22833200 -1.9262460 0.52372200

Au 2.06747800 0.07190000 0.18815500

Cl 0.90385000 2.08144600 -0.10900700

Cl 6.64257100 0.99576700 -0.52610000

Au -0.78868500 0.47649500 -0.13796300

Au 4.94087800 -0.4083910 -0.01546500

Cl -2.44528800 -1.1477800 -0.23013100

Cl -6.69995200 0.66515400 0.21754900

Au -4.60040300 -0.1960380 -0.00409700

Cl -5.47338100 -2.2944030 -0.38148500

Cl -3.68096600 1.88645700 0.363116000
